# Dual-Side Hybrid Embedding Network for Gain Enhancement of Terahertz Amplifiers at Frequencies Near *f*_max_

**DOI:** 10.3390/mi17040432

**Published:** 2026-03-31

**Authors:** Xiaorui Liu, Jianguo Yu, Yun Wang, Yibo Huang, Feixiang Zhang, Zhanjiang Wang, Yaqi Cheng

**Affiliations:** School of Electronic Engineering, Beijing University of Posts and Telecommunications, Beijing 100876, China; lxr1100@bupt.edu.cn (X.L.);

**Keywords:** embedding network, gain enhancement, terahertz amplifier, defected ground structure (DGS)

## Abstract

This paper proposes a dual-side hybrid embedding network (DHEN) to mitigate gain degradation in terahertz amplifiers at frequencies near $f_{\max}$. The proposed approach employs a pre-embedding network for parasitic absorption, followed by Y-embedding for gain enhancement. A theoretical analysis is conducted to derive the embedding conditions for process-constrained circuit synthesis. In this architecture, a capacitive base-side pre-embedding provides intrinsic DC isolation, while a defected-ground-structure (DGS) inductor realizes the Y-embedding inductive element with reduced layout area. Based on the DHEN, a four-stage amplifier is designed in a 130 nm SiGe BiCMOS process. Electromagnetic co-simulation results demonstrate a power gain of 19.3 dB at 280 GHz, corresponding to an 11.5 dB improvement over a conventional unboosted amplifier. The proposed approach provides a unified synthesis methodology that simultaneously addresses parasitic absorption, DC isolation, and gain enhancement for near-$f_{\max}$ THz amplifier design.

## 1. Introduction

Terahertz (THz) technology has demonstrated significant potential in applications such as radio astronomy, high-resolution imaging, and high-speed wireless communications, owing to its high data capacity, enhanced security, and high atmospheric transparency in specific spectral windows [1,2,3,4,5,6,7]. However, THz systems suffer from severe free-space path loss and atmospheric attenuation, which necessitate multiple gain stages to achieve long-range wireless transmission [8,9,10,11,12,13]. These gain stages must employ active devices with sufficiently high maximum oscillation frequency ($f_{\max}$) to operate effectively at THz frequencies [14,15].

Nevertheless, as the operating frequency approaches the transistor $f_{\max}$, the intrinsic gain of the active device decreases rapidly, particularly beyond half $f_{\max}$ [16,17,18,19,20,21,22,23,24,25,26,27,28,29,30]. Consequently, silicon-based THz amplifiers without gain enhancement techniques exhibit insufficient power gain to meet system requirements. One straightforward approach is to cascade multiple stages, which, however, leads to increased chip area and DC power consumption. Alternatively, positive feedback (PF) techniques can be employed to enhance the gain of each amplifier stage. Singhakowinta [21] demonstrated that embedding an active two-port network (A2P) within a linear lossless reciprocal (LLR) network can increase the maximum available gain ($G_{ma}$) under conjugate matching while maintaining unconditional stability (K≥1). This process continues until $G_{ma}$ reaches the theoretical upper bound $G_{max}=\left(2U-1\right)+2\sqrt{U(U-1)}$, where *U* denotes the unilateral gain [27]. Based on this principle, various gain enhancement strategies have been reported, including parallel (Y-) embedding, series (Z-) embedding, and their corresponding pre-embedding configurations. These methods enable device gain to approach $G_{max}$ at frequencies near half $f_{\max}$ [22,23,24,25,26,27,28,29].

Despite their effectiveness, conventional LLR-based gain enhancement structures often lack intrinsic DC-blocking capability, resulting in constrained biasing flexibility. In particular, enforcing equal base and collector voltages may degrade key performance metrics [30]. Beyond the DC-blocking issue, the key practical challenge is to jointly handle parasitic absorption, gain enhancement, and process constraints in a unified synthesis flow for near-$f_{\max}$ amplifier design.

To address these limitations, this paper proposes a dual-side hybrid embedding network (DHEN) for gain enhancement in near-$f_{\max}$ THz amplifiers. The DHEN employs a base-side capacitive pre-embedding network and a collector-side Y-embedding network to absorb parasitics, enhance gain, and maintain inherent DC isolation for independent bias control. Unlike prior LLR-based and lossy embedding approaches, the proposed DHEN unifies parasitic absorption and DC isolation within a single synthesis framework, representing the key novelty of this work. A compact DGS inductor is adopted to implement the inductive embedding with reduced layout area. This work develops the analytical framework and synthesis methodology of the DHEN under process constraints, with all passive components EM-modeled and co-simulated with device models. The remainder of this paper is organized as follows. Section 2 introduces the DHEN structure and theoretical analysis; Section 3 presents the amplifier design and simulation results; Section 4 concludes this paper.

## 2. DHEN Gain Enhancement Technique

Different from conventional dual-side embedding approaches that rely solely on inductive elements, the proposed DHEN introduces a base-side pre-embedding that simultaneously realizes the required reactive embedding and intrinsic DC isolation. This integration enables gain-plane synthesis under explicit bias constraints, rather than treating biasing as an external design consideration.

### 2.1. Y-Matrix Analysis of the DHEN

**Figure 1:**
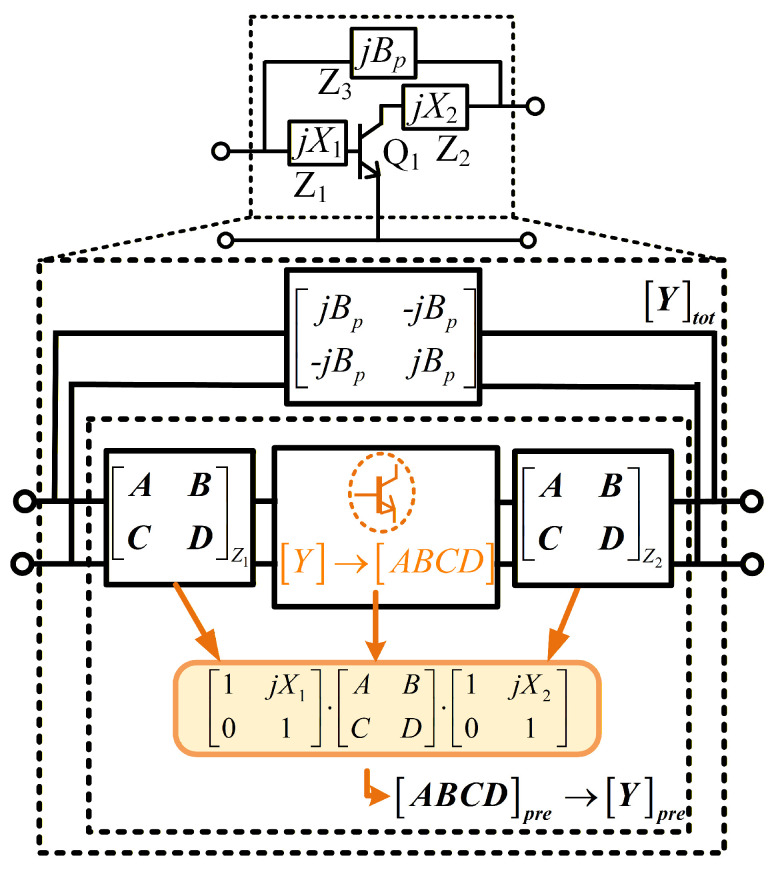
Analytical representation of the proposed DHEN using ABCD- and Y-parameter formulations.

To analyze the proposed DHEN structure, the Y-matrix representation of each sub-network is first examined. Figure 1 illustrates the analytical representation of the DHEN, where reactive elements $jX_{1}$ and $jX_{2}$ form the pre-embedding network and a shunt susceptance $B_{p}$ realizes the Y-embedding network.

The transistor Q1 is modeled as a two-port network characterized by its Y-matrix $\left[Y\right]=\left[Y_{11},Y_{12};Y_{21},Y_{22}\right]$. This Y-matrix can be equivalently transformed into the corresponding ABCD-matrix [ABCD]. By inserting these reactive elements at both ports of the A2P, a pre-embedding network is formed. The resulting ABCD-matrix of the pre-embedding network can be written as

(1) $$\begin{array}{cc} {\left[ABCD\right]}_{pre} & ={\left[\begin{array}{cc} A & B\\ C & D \end{array}\right]}_{Z_{1}}\cdot \left[\begin{array}{cc} A & B\\ C & D \end{array}\right]\cdot {\left[\begin{array}{cc} A & B\\ C & D \end{array}\right]}_{Z_{2}}\\ \; & =\left[\begin{array}{cc} 1 & jX_{1}\\ 0 & 1 \end{array}\right]\cdot \left[\begin{array}{cc} -\frac{Y_{22}}{Y_{21}} & -\frac{1}{Y_{21}}\\ -\frac{\Delta Y}{Y_{21}} & -\frac{Y_{11}}{Y_{21}} \end{array}\right]\cdot \left[\begin{array}{cc} 1 & jX_{2}\\ 0 & 1 \end{array}\right]\\ \; & =-\frac{1}{Y_{21}}\left[\begin{array}{cc} jX_{1}\cdot \Delta Y+Y_{22} & M_{pre}\\ \Delta Y & jX_{2}\cdot \Delta Y+Y_{11} \end{array}\right] \end{array}$$

where $\Delta Y=Y_{11}Y_{22}-Y_{12}Y_{21}$ and $M_{pre}=1+jX_{1}Y_{11}+jX_{2}Y_{22}-X_{1}X_{2}\Delta Y$.

From (1), the corresponding Y-matrix of the pre-embedding network ${\left[Y\right]}_{pre}$ can be derived as

(2) $${\left[Y\right]}_{pre}=\frac{1}{M_{pre}}\left[\begin{array}{cc} jX_{2}\cdot \Delta Y+Y_{11}\; & Y_{12}\\ Y_{21}\; & jX_{1}\cdot \Delta Y+Y_{22} \end{array}\right].$$

As illustrated in Figure 1, the complete DHEN is obtained by shunt-connecting a Y-embedding network at both the input and output ports of the pre-embedding network. The resulting overall Y-matrix is given in (3)

(3) $$\begin{array}{cc} {\left[Y\right]}_{tot} & ={\left[Y\right]}_{pre}+\left[\begin{array}{cc} jB_{p} & -jB_{p}\\ -jB_{p} & jB_{p} \end{array}\right]\\ \; & =\left[\begin{array}{cc} \frac{jX_{2}\cdot \Delta Y+Y_{11}}{M_{pre}}+jB_{p} & \frac{Y_{12}}{M_{pre}}-jB_{p}\\ \frac{Y_{21}}{M_{pre}}-jB_{p} & \frac{jX_{1}\cdot \Delta Y+Y_{22}}{M_{pre}}+jB_{p} \end{array}\right]. \end{array}$$

Based on the total Y-matrix ${\left[Y\right]}_{tot}$, the influence of the DHEN on the original A2P can be conveniently visualized and analyzed on the gain plane.

### 2.2. Gain-Plane-Based Analytical Solution of the DHEN

**Figure 2:**
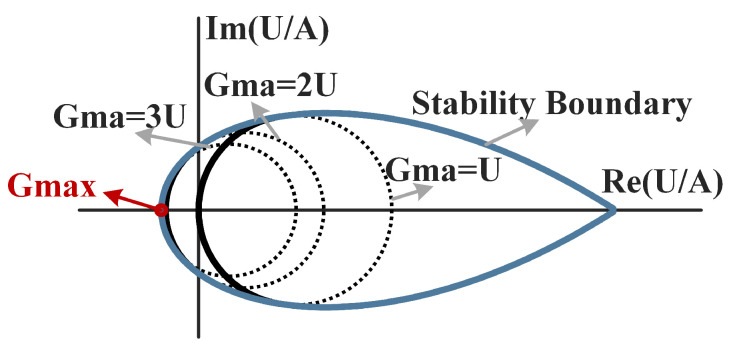
Gain plane and gain circles.

The gain plane is a graphical tool used to analyze the influence of embedding networks on $G_{ma}$ and *U*, as shown in Figure 2 [21]. On the gain plane, the transistor characteristics, including $G_{ma}$ and the stability factor *K*, can be mapped onto a two-dimensional coordinate system defined by the ratio between *U* and the gain parameter *A*, where $A=Y_{21}/Y_{12}$. In this representation, constant gain values correspond to specific gain circles, while the stability boundary separates the unconditionally stable region (K≥1) from the unstable region. Since *U* remains invariant for all LLR networks added to the A2P, variations in $G_{ma}$ and *K* depend solely on *A*. Consequently, the movement of the operating point on the gain plane directly reflects the impact of external network tuning on the overall performance.

According to [22], the *A* of the overall network is given by

(4) $$A_{\mathrm{tot}}=\frac{Y_{21\mathrm{tot}}}{Y_{12\mathrm{tot}}}=\frac{Y_{21}-jB_{p}\cdot M_{pre}}{Y_{12}-jB_{p}\cdot M_{pre}}.$$

Since all embedding networks are LLRs, the value of *U* remains constant; therefore, the gain-state point of the overall network becomes

(5) $$\frac{U}{A_{tot}}=\frac{U(Y_{12}-jB_{p}\cdot M_{pre})}{Y_{21}-jB_{p}\cdot M_{pre}}.$$

As can be observed from (5), the pre-embedding network itself does not directly shift the gain-state point. Instead, the displacement of the gain-state point is induced by the Y-embedding network. The introduction of the pre-embedding network increases the degrees of freedom governing the trajectory of the gain-state point on the gain plane from a single variable $B_{p}$ to a combined parameter set $\left(X_{1},X_{2},B_{p}\right)$, thereby enabling more flexible structural optimization.

Based on the gain-plane method, the following conditions must be satisfied to achieve $G_{\max}$ [27]:

(6) ReUAtot=−U2U−1+2U(U−1)ImUAtot=0

By substituting (5) into (6), an infinite set of feasible embedding-element combinations can be obtained, since the number of design variables exceeds that of independent equations. By fixing $X_{2}$, the remaining parameters $X_{1}$ and $B_{p}$ can be expressed as follows:

(7) $$\left\{\begin{array}{cc} B_{p} & =\frac{m_{2}m_{5}-m_{4}m_{6}}{m_{1}m_{4}+m_{2}m_{3}}\\ X_{1} & =\frac{m_{5}-m_{3}B_{p}}{m_{4}B_{p}} \end{array},\right.$$

where

(8) m1=1−X2·Im(Y22)·(a−1)m2=Im(Y11)+X2·Re(ΔY)·(a−1)m3=X2·Re(Y22)·(a−1)m4=Re(Y11)−X2·Im(ΔY)·(a−1)m5=Re(Y12)−a·Re(Y21)m6=Im(Y12)−a·Im(Y21)a=−12U−1+2U(U−1)

For a given value of $X_{2}$, the corresponding values of $X_{1}$ and $B_{p}$ can be determined using (7).

## 3. Design Examples

### 3.1. Amp-Cell

In this section, a representative amp-cell design example is presented to validate the analytical evaluation of the proposed DHEN-based gain enhancement technique under process constraints. The circuit is designed in a 130 nm SiGe BiCMOS process (with $f_{\max}=450$ GHz), and the target operating frequency is set to 280 GHz, which lies beyond half of $f_{\max}$. The same design kit, as well as a comparable frequency range, has been extensively characterized and experimentally validated in prior works [17,18,19,20]. In this process, 280 GHz corresponds to approximately $0.62f_{\max}$, a regime in which meaningful transistor gain remains available; the practical feasibility of 280 GHz operation in 130 nm SiGe BiCMOS is strongly supported by measured demonstrations across the 206–310 GHz range from these cited works.

In the design of the proposed DHEN, the base-side pre-embedding is formed by a capacitor. This choice inherently provides DC isolation while participating in the reactive embedding required by the analytical synthesis, thereby enabling a direct mapping between the theoretical formulation and circuit-level realization.

**Figure 3:**
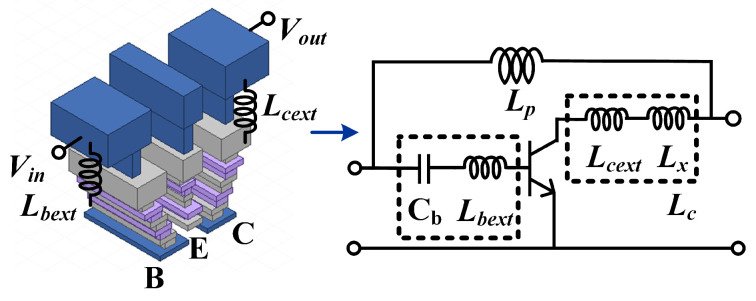
Metal interconnect and associated parasitic inductances, where $L_{bext}$ and $L_{cext}$ denote the extracted base-side and collector-side parasitic inductances, respectively.

The employed HBT features an emitter area of $A_{E}=4\times 0.07\times 0.9$ μm^2^ and is biased at $V_{b}=0.94$ V and $V_{cc}=1.5$ V. At THz frequencies, parasitic effects associated with passive interconnects become non-negligible and must be carefully incorporated into the design procedure. In particular, as illustrated in Figure 3, the parasitic inductance between the HBT base port (M1) and the extrinsic top-metal connection (TM2) is extracted to be approximately 4.9 pH at 280 GHz. The extraction is performed from the EM-simulated interconnect admittance using Lext=Imy11−1ω [31], where $y_{11}$ is the interconnect admittance obtained from EM simulation. These parasitics are explicitly included in the pre-embedding network to ensure consistency between the analytical evaluation and the circuit-level design.

**Figure 4:**
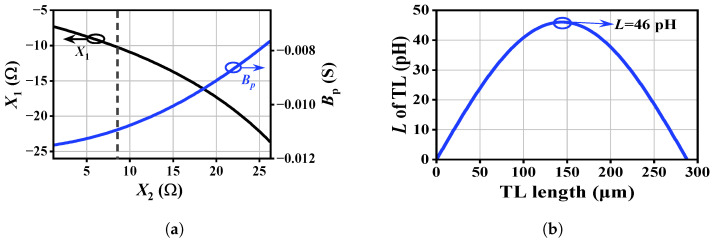
Embedding network solutions and transmission-line realization: (**a**) $X_{1}$ (black), $B_{p}$ (blue), and the selected process-constrained solution (dashed line). (**b**) Equivalent inductance versus TL length at 280 GHz with $Z_{0}=81\;\Omega$; arrow marks the maximum realizable inductance (≈46 pH).

By sweeping $X_{2}$ from 1 Ω to 27 Ω, the corresponding values of $X_{1}$ and $B_{p}$ were solved from (7) and are plotted in Figure 4a. To mitigate high-frequency loss, the Y-embedding inductance $L_{p}$ should be minimized. As shown in Figure 4a, decreasing the collector-side reactance $X_{2}$ reduces the Y-embedding susceptance $B_{p}$, thereby yielding a smaller $L_{p}$. Since the collector is biased through the top-metal layer, a purely inductive element is preferred at this node. Consequently, the minimum practical value of the collector embedding element is limited by its constrained parasitic inductance $L_{cext}$, corresponding to $X_{2}\approx 8.62\;\Omega$ at 280 GHz. Under this condition, the resulting parameters are $X_{1}=-10.27\;\Omega$ and $B_{p}=-0.0109\;S$. After absorbing the base parasitic inductance $L_{bext}$, $X_{1}$ translates into an additional 30 fF series capacitor, while $B_{p}$ corresponds to a 52 pH inductor at 280 GHz.

After determining the initial reference values of the embedding network, further optimization is required to account for process constraints. Under these constraints, parameters such as the minimum line width directly limit the maximum achievable characteristic impedance and the realizable inductance range of transmission lines (TL). When the TL length is shorter than half the wavelength (λ/2), parasitic shunt components can be neglected, and the line can be approximated as a pure inductor, whose inductance satisfies $\omega L_{M}=Z_{0}\sin\left(\beta l\right)$.

Figure 4b illustrates the relationship between the equivalent inductance and the physical length of the TL at 280 GHz. As shown in Figure 4b, the maximum inductance achievable with TL under the given process constraints is approximately 46 pH, which is lower than the required Y-embedding inductance of 52 pH obtained from (7). This discrepancy indicates that further optimization is necessary; consequently, the Y-embedding inductance derived from Figure 4a cannot be directly synthesized using conventional TL under the given constraints.

**Figure 5:**
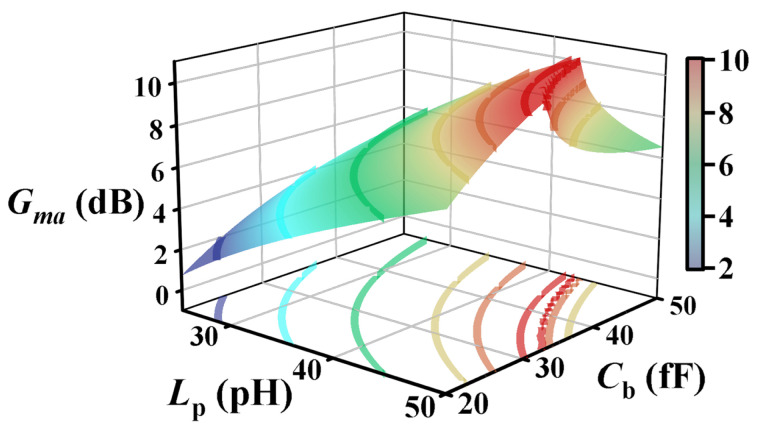
$G_{ma}$ as a function of $C_{b}$ and $L_{p}$ in the Amp-cell ($L_{c}=4.9$ pH).

To overcome this process limitation, a re-optimization is performed by adjusting the base-side pre-embedding capacitance $C_{b}$, which is initially determined from the analytical solution. By increasing $C_{b}$, the required value of the parallel embedding inductance $L_{p}$ can be effectively relaxed while preserving the targeted gain performance. Figure 5 illustrates the variation of $G_{ma}$ in the Amp-cell as a function of $C_{b}$ and $L_{p}$ for a fixed collector inductance of $L_{c}=4.9$ pH. As shown in Figure 5, for a given gain level, the required Y-embedding inductance $L_{p}$ decreases with increasing $C_{b}$, enabling a practical re-selection of the embedding parameters within the feasible design space. Accordingly, the final values are chosen as $L_{p}=44.5$ pH and $C_{b}=43$ fF.

**Figure 6:**
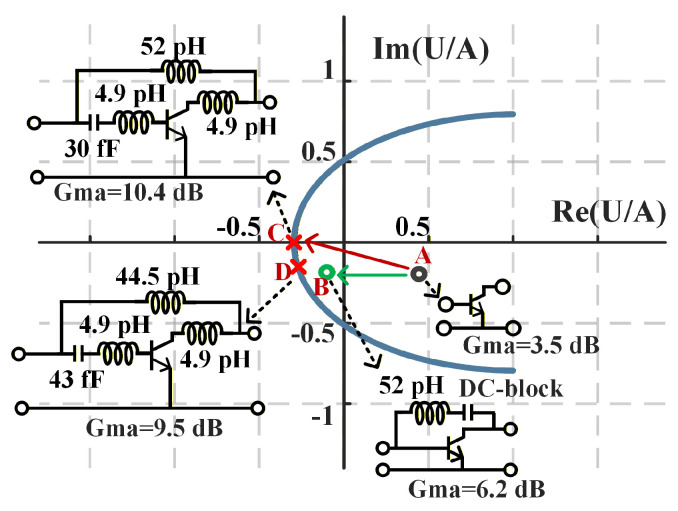
The trajectory of the gain-state point of the Amp-cell at 280 GHz under different embedding conditions: A, original A2P without embedding; B, Y-embedding only; C, ideal DHEN solution reaching $G_{max}$; D, process-constrained DHEN solution.

The trajectory of the gain-state point of the Amp-cell at 280 GHz under different embedding conditions is shown in Figure 6. Point A corresponds to the original A2P without any embedding network, yielding a $G_{ma}$ of 3.5 dB, which is far below $G_{max}$. Point B represents the gain-state point with only the Y-embedding network applied, where $G_{ma}$ increases to 6.2 dB and the trajectory moves toward $G_{max}$ yet does not reach it. This behavior highlights the necessity of introducing a pre-embedding network to further steer the gain-state trajectory. Point C corresponds to the theoretical DHEN solution with pre-embedding included, for which the gain-state point reaches $G_{max}$ (10.4 dB), resulting in an overall gain improvement of approximately 6.9 dB compared to the original A2P. Point D denotes the DHEN solution under process constraints, achieving a $G_{ma}$ of 9.5 dB, which is 0.9 dB lower than $G_{max}$ due to process constraints.

**Figure 7:**
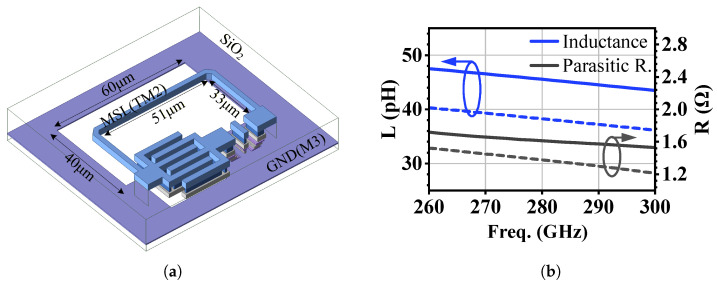
DGS inductor: (**a**) three-dimensional view; (**b**) simulated inductance and parasitic resistance of the DGS (solid lines) and solid-ground (dashed lines) inductors.

Although the process-constrained DHEN solution (Point D in Figure 6) already provides a substantial gain improvement, its design still requires a compact embedding inductor with sufficiently high inductance density. To address this requirement, a defected-ground-structure (DGS) inductor is introduced to enhance the achievable inductance within a limited footprint. Figure 7a shows the three-dimensional view of the DGS inductor, in which a microstrip line (MSL) on the TM2 metal layer is routed above a patterned ground plane on the M3 metal layer. By introducing intentional defects in the ground plane beneath the signal line, the electromagnetic field distribution is perturbed, leading to an increased equivalent inductance compared with a solid-ground inductor. Figure 7b compares the simulated inductance and parasitic resistance of the proposed DGS inductor (solid lines) with those of a conventional solid-ground inductor (dashed lines). As shown, the DGS inductor exhibits a higher equivalent inductance over the frequency range of interest, achieving approximately 44 pH at the target frequency. In contrast, the solid-ground inductor provides about 38 pH under the same physical dimensions. Meanwhile, the DGS structure results in a higher parasitic resistance, reflecting the increased loss associated with the ground-plane defects.

**Figure 8:**
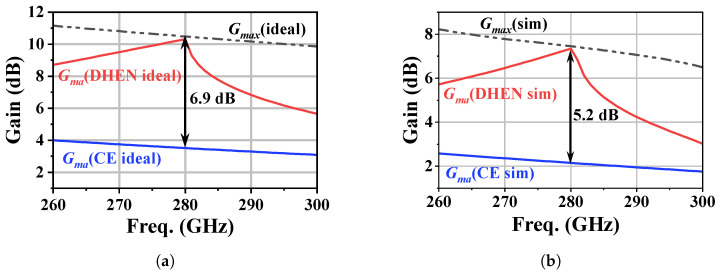
Comparison of $G_{ma}$ between traditional CE and DHEN architectures: (**a**) ideal lossless embedding network; (**b**) EM-simulation-based embedding network.

After EM simulation, an equivalent inductance of approximately 44 pH is obtained at 280 GHz using the DGS inductor shown in Figure 7a, with a total effective length of about 118 μm. A 43 fF cross-finger capacitor with a Q of 15 is realized with two thick metal layers. Figure 8 compares the maximum available gain $G_{ma}$ of the conventional common-emitter (CE) amplifier with that of the DHEN-based design. In Figure 8a, where parasitic effects are not considered, the theoretical upper bound of the gain, $G_{max}$, is 10.4 dB at 280 GHz. Under the same condition, the conventional CE amplifier exhibits a maximum available gain $G_{ma}$ of only 3.5 dB, which is well below this upper bound. With the introduction of the DHEN, $G_{ma}$ is increased from 3.5 dB to 10.4 dB, reaching the theoretical upper bound and corresponding to a gain enhancement of approximately 6.9 dB per stage. Here, the “ideal” case in Figure 8a does not include transistor interconnect parasitics. In Figure 8b, where parasitic effects are included through EM simulation, the theoretical upper bound $G_{max}$ is reduced to 7.3 dB. Nevertheless, the DHEN remains effective, improving $G_{ma}$ from 2.1 dB to 7.1 dB and yielding a gain enhancement of about 5 dB. The gain reduction from the ideal case (about 3.3 dB) is mainly attributed to parasitic effects of the transistor peripheral interconnects and the passive/layout loss of the realized DHEN network; the CE reference is treated under the same comparison setup.

### 3.2. Multi-Stage Amplifier Design

**Figure 9:**
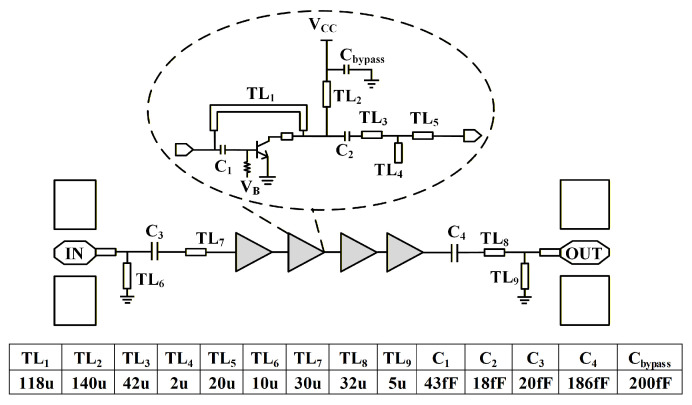
The schematic of the four-stage amplifier is based on Amp-cell.

A four-stage amplifier is designed based on the Amp-cell introduced in Section 3.1, as illustrated in Figure 9. All transmission lines used for impedance matching in Figure 9 are realized using 56 Ω MSLs, with metal layer M3 serving as the reference ground plane and TM2 as the signal layer. A 140 μm MSL is employed for DC decoupling to provide RF isolation between the bias network and the signal path. To mitigate gain roll-off at higher frequencies, inter-stage impedance matching is optimized at 285 GHz, thereby maintaining a relatively flat gain response around the target operating frequency.

**Figure 10:**
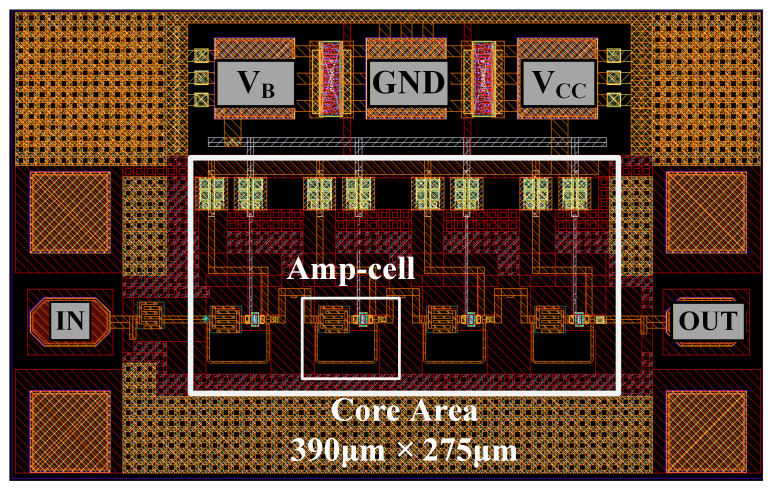
Layout view of the designed four-stage amplifier.

The layout of the four-stage amplifier is shown in Figure 10. The core occupies an area of 390 μm × 275 μm.

**Figure 11:**
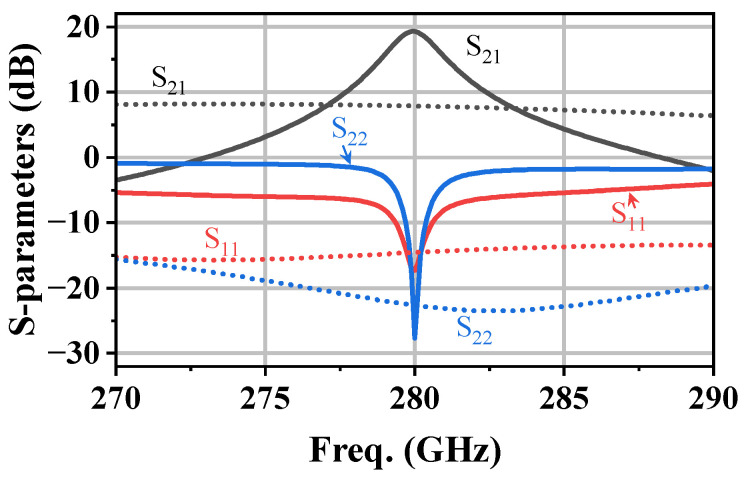
Simulated S-parameters of the proposed DHEN-based four-stage amplifier (solid) and the conventional CE amplifier (dashed), with all passive components EM-modeled.

The circuit shown in Figure 9 was simulated, where all passive components, including MSLs and capacitors, were modeled using an EM simulator to account for parasitic effects. As shown in Figure 11, the amplifier achieves an $S_{21}$ of 19.3 dB at 280 GHz, making it suitable for use as a driver amplifier in communication links. To verify the effectiveness of the proposed architecture, a conventional four-stage CE amplifier without any gain enhancement technique was also designed as a reference. This reference amplifier exhibits an $S_{21}$ of only 7.8 dB at 280 GHz. The overall gain improvement in the four-stage amplifier is lower than the ideal per-stage enhancement because cumulative inter-stage matching and passive-network losses are included in the EM-co-simulated multi-stage design. Both amplifiers are well matched, with input/output return losses better than 15 dB. These results confirm that the proposed DHEN technique effectively enhances the power gain of amplifiers operating near $f_{max}$.

**Figure 12:**
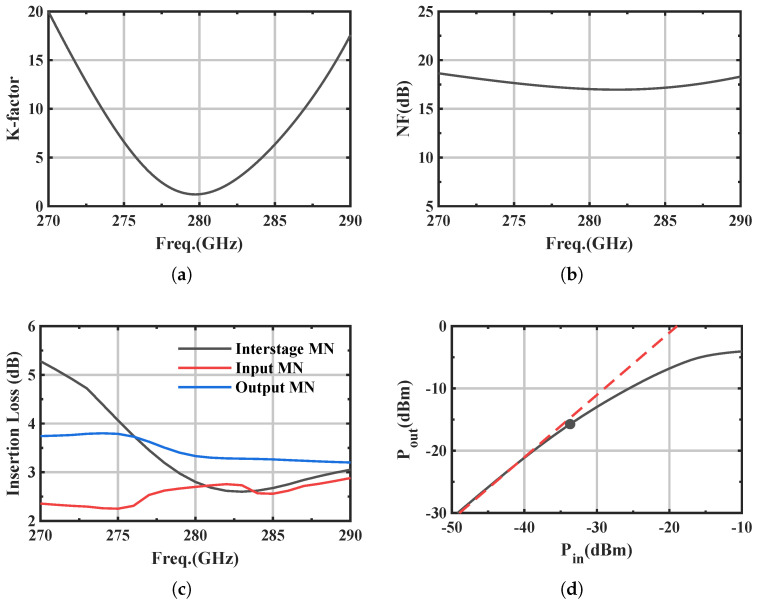
Simulated performance summary of the proposed DHEN-based four-stage amplifier: (**a**) stability factor *K*; (**b**) noise figure; (**c**) insertion loss of key passive matching networks; (**d**) large-signal $P_{\mathrm{out}}$–$P_{\mathrm{in}}$ characteristic, where the solid curve denotes the simulated large-signal response, the dashed line denotes the linear extrapolation, and the dot marks the output 1 dB compression point.

To provide a more complete performance characterization of the proposed four-stage amplifier, additional simulated results are summarized in Figure 12, including stability around the design band, noise figure, passive-network insertion loss, and large-signal output-power behavior.

Figure 12a indicates that *K* remains above 1 (minimum ≈1.1 near 280 GHz), confirming operation in the unconditionally stable region (K≥1) defined in Section 2.2. The minimum simulated noise figure is about 16.9 dB near 282 GHz, as shown in Figure 12b. Figure 12c summarizes the insertion loss of key passive networks: the inter-stage matching network shows the largest and most frequency-dependent loss, decreasing from about 5.3 dB at 270 GHz to about 2.6 dB near 290 GHz, while the input/output matching losses vary more mildly. Figure 12d presents the large-signal $P_{\mathrm{out}}$–$P_{\mathrm{in}}$ characteristic, from which the output 1 dB compression point and saturation power are approximately −16 dBm and −4 dBm, respectively.

To further benchmark the proposed design, Table 1 compares this work with representative silicon-based sub-THz amplifiers reported near the target frequency range. As shown in Table 1, this work achieves the highest gain per stage among the listed designs (4.83 dB/stage at 280 GHz, simulated), indicating strong gain-enhancement capability under near-$f_{\max}$ operation. The 11.5 dB total gain improvement over the unboosted CE reference—obtained within the same process and at the same frequency—is therefore significant: within the compared set of silicon-based sub-THz demonstrations (Table 1, noting differences in process node and topology), it achieves the highest per-stage gain and directly validates the gain-enhancement efficacy of the proposed DHEN methodology.

## 4. Conclusions

This paper presents a gain enhancement approach for amplifiers operating near the transistor $f_{\max}$ based on a dual-side hybrid embedding network. Closed-form expressions derived from gain-plane theory provide analytical guidance for embedding synthesis. The proposed DHEN absorbs interconnect parasitics while a capacitive base-side pre-embedding enables independent bias control without additional DC-blocking capacitors. A four-stage amplifier designed in a 130 nm SiGe BiCMOS process achieves a power gain of 19.3 dB at 280 GHz, corresponding to an 11.5 dB improvement over a conventional unboosted CE amplifier, while maintaining stable operation. These results—fully consistent with the abstract claims—confirm that the proposed DHEN technique effectively enhances the gain of amplifiers operating near $f_{max}$. It should be noted that this work primarily focuses on validating the upper-limit gain enhancement at the designated design frequency under near-$f_{\max}$ operation. Accordingly, the optimization prioritizes peak gain at the target frequency, while gain–bandwidth co-optimization is beyond the main scope of this paper. Future work will build on the current high-gain core to pursue bandwidth extension, thereby improving usable bandwidth and practical applicability.

## Figures and Tables

**Table 1:** Comparison of representative silicon-based sub-THz amplifiers near the target frequency range.

Ref.	Technology	Frequency (GHz)	$f_{\max}$ (GHz)	$f/f_{\max}$	Gain (dB)	Gain/Stage (dB)
[22]	65 nm CMOS	260	352	0.74	9.2	2.3
[25]	65 nm CMOS	280	395	0.71	12	4
[30]	65 nm CMOS	298	317	0.94	21	1.31
[32]	130 nm SiGe	283	450	0.63	10.9	0.78
This work	130 nm SiGe	280 *	450	0.62	19.3 *	4.83

* Simulated.

## Data Availability

Data underlying the results presented in this paper are not publicly available at this time but may be obtained from the authors upon reasonable request.
